# Supplementary material for: Identification of Freezing-Responsive microRNAs and Their Targets in Chinese Jujube by Small RNA and Degradome Sequencing
Source: Int J Mol Sci. 2026 Sep 4;27(17):7912. doi: 10.3390/ijms27177912 (PMC13566890; doi:10.3390/ijms27177912)
Supplement: Supplementary file 1 [file ijms-27-07912-s001.zip › Supplementary Material S2/novel_miR_104.pdf]

Provisional ID	:	novel_miR_104
Score total	:	0.4
Score for star read(s)	:	-1.3
Score for read counts	:	0
Score for mfe	:	0.1
Score for randfold	:	1.6
Score for cons. seed	:	
Total read count	:	594
Mature read count	:	594
Loop read count	:	0
Star read count	:	0

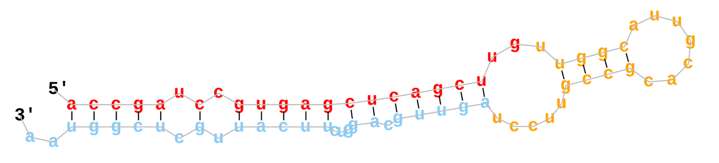

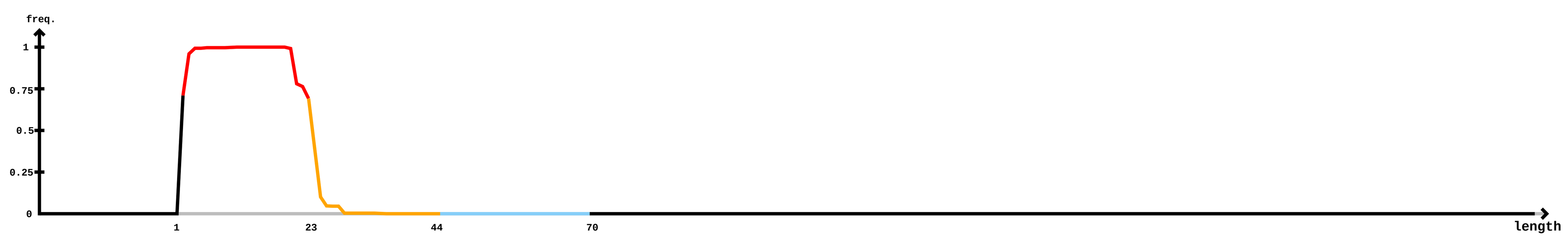

Mature Star

```
5'- uuguucauuuaguaacguauuuaccgauccgugagcucagcuuguuggcauugcacgccguuccuaguugcaggucuucauugcucgguaaaccaaaaaauagcaauguauuuuauuaauuuuauuugauaaaauuuauuaauaucacuuuugaugagacauaaauuaaacaagucuauuuaauuagauuuuugacaauaaauugucaauuaauuaacuuuugacaacuugcauauauuugucguuguac -3'   exp
    (((((.(((((((..(((.((((((((.(.(((((((((((...((((.......)))).....))))).))...))))).).)))))))).(((((.(((..((((.((((((((((......)))))))))).))))..)))...))))))))..)).)))))..)))))(((((......)))))..(((((((....))))))).............(((((..(((......))).)))))..   reads   mm   sample
    ......................accgauccgugagcucag..................................................................................................................................................................................................   1   0   LT-2
    ......................accgauccgugagcucag..................................................................................................................................................................................................   3   0   LT-3
    ......................accgauccgugagcucag..................................................................................................................................................................................................   1   0   LT-1
    ......................accgauccgugagcucagc.................................................................................................................................................................................................   6   0   JCK-3
    ......................accgauccgugagcucagc.................................................................................................................................................................................................   15   0   JCK-2
    ......................accgauccgugagcucagc.................................................................................................................................................................................................   6   0   LT-1
    ......................accgauccgugagcucagc.................................................................................................................................................................................................   4   0   JT-2
    ......................accgauccgugagcucagc.................................................................................................................................................................................................   5   0   JCK-1
    ......................accgauccgugagcucagc.................................................................................................................................................................................................   6   0   JT-3
    ......................accgauccgugagcucagc.................................................................................................................................................................................................   2   0   LCK-3
    ......................aNcgauccgugagcucagc.................................................................................................................................................................................................   1   1   JCK-2
    ......................accgauccgugagcucagc.................................................................................................................................................................................................   9   0   LT-3
    ......................accgauccgugagcucagc.................................................................................................................................................................................................   20   0   JT-1
    ......................accgauccgugagcucagc.................................................................................................................................................................................................   6   0   LT-2
    ......................accgauccgugagcucagcu................................................................................................................................................................................................   3   0   LT-3
    ......................accgauccgugaCcucagcu................................................................................................................................................................................................   1   1   LT-2
    ......................accgauccgugagcucagcu................................................................................................................................................................................................   2   0   LT-1
    ......................accgauccgugagcucagcu................................................................................................................................................................................................   3   0   LT-2
    ......................accgauccgugagcucagcuu...............................................................................................................................................................................................   9   0   JCK-3
    ......................accgauccgugagcucagcuu...............................................................................................................................................................................................   9   0   JCK-2
    ......................accgauccgugagcucagcuu...............................................................................................................................................................................................   3   0   LT-2
    ......................accgauccgugagcucagcuu...............................................................................................................................................................................................   3   0   LT-1
    ......................accgauccgugagcucagcuu...............................................................................................................................................................................................   2   0   LT-3
    ......................accAauccgugagcucagcuu...............................................................................................................................................................................................   1   1   JCK-3
    ......................accgauccgugagcucagcuu...............................................................................................................................................................................................   9   0   JCK-1
    ......................accgauccgugagcucagcuuU..............................................................................................................................................................................................   1   1   LT-1
    ......................accgauccgugagcucagcuug..............................................................................................................................................................................................   28   0   JCK-1
    ......................acNgauccgugagcucagcuug..............................................................................................................................................................................................   1   1   LT-2
    ......................Cccgauccgugagcucagcuug..............................................................................................................................................................................................   1   1   LCK-1
    ......................accgauccgugagcucagcuuU..............................................................................................................................................................................................   4   1   JCK-2
    ......................accgauccgugagcucCgcuug..............................................................................................................................................................................................   2   1   LCK-1
    ......................accgauccgugagcucagcuuC..............................................................................................................................................................................................   1   1   JCK-2
    ......................accgauccgugagcucagcuug..............................................................................................................................................................................................   10   0   JT-1
    ......................accgauccgugagcucagcuug..............................................................................................................................................................................................   10   0   LT-2
```

```
                      Mature                   Star
uuguucauuuaguaacguauuuaccgauccgugagcucagcuuguuggcauugcacgccguuccuaguugcaggucuucauugcucgguaaaccaaaaaaauagcaauguauuuuauuaauuuuauuugauaaaauuuauuaauaucacuuuugaugagacauaaauuaaacaagucuauuuaauuagauuuuugacaauaaauugucaauuaauuaacuuuugacaacuugcauauauuugucguuguac
......................accgauccgugagcucagcuug...............................................................................................................................................................................   61   0   JCK-2
......................accgauccgugagcucagcuug...............................................................................................................................................................................   9    0   LT-3
......................accgaucUgugagcucagcuug...............................................................................................................................................................................   2    1   JCK-1
......................accgauccgugagcucagcuuU...............................................................................................................................................................................   3    1   JCK-1
......................accgauccgugagcucagcuug...............................................................................................................................................................................   26   0   JCK-3
......................accgauccgugagcucagcuug...............................................................................................................................................................................   26   0   LCK-2
......................accgauccgugagcucagcuug...............................................................................................................................................................................   37   0   LCK-1
......................Cccgauccgugagcucagcuug...............................................................................................................................................................................   1    1   LT-3
......................accgauccgugagcucagcuug...............................................................................................................................................................................   7    0   LT-1
......................accgauccgugagcucagcuug...............................................................................................................................................................................   3    0   JT-2
......................accgCuccgugagcucagcuug...............................................................................................................................................................................   1    1   LCK-1
......................accgauccgugagcucCgcuug...............................................................................................................................................................................   2    1   LCK-2
......................accgauccgugaNcucagcuug...............................................................................................................................................................................   1    1   LCK-1
......................accgauccgugagcucagcuuU...............................................................................................................................................................................   2    1   JCK-3
......................accgauccgugagcucagcuug...............................................................................................................................................................................   4    0   JT-3
......................accgauccgugagcucagcuugu..............................................................................................................................................................................   2    0   JCK-3
......................accgauccgugagcucagcuugu..............................................................................................................................................................................   4    0   LT-3
......................accgauccgugagcucagcuugu..............................................................................................................................................................................   1    0   JCK-1
......................aNcgauccgugagcucagcuugu..............................................................................................................................................................................   1    1   JCK-3
......................accgauccgugagcucagcuugu..............................................................................................................................................................................   4    0   JCK-2
......................accgauccgugagcucagcuugu..............................................................................................................................................................................   7    0   LCK-3
......................accgauccgugagcucagcuuguA.............................................................................................................................................................................   2    1   JCK-2
......................accgauccgugagcucagcuuguA.............................................................................................................................................................................   1    1   JCK-1
......................accgauccgugagcucagcuuguu.............................................................................................................................................................................   2    0   JCK-3
......................accgauccgugagcucagcuuguA.............................................................................................................................................................................   1    1   LT-1
......................accgauccgugagcucagcuuguu.............................................................................................................................................................................   9    0   JCK-1
......................accgauccgugagcucagcuuguA.............................................................................................................................................................................   1    1   JCK-3
......................accgauccgugagcucagcuuguu.............................................................................................................................................................................   5    0   LT-2
......................accgauccgugagcucagcuuguu.............................................................................................................................................................................   6    0   JCK-2
......................accgauccgugagcucagcuuguC.............................................................................................................................................................................   2    1   JCK-3
......................accgauccgugagcucagcuuguug............................................................................................................................................................................   1    0   LT-3
......................accgauccgugagcucagcuuguuggc..........................................................................................................................................................................   1    0   LT-1
.......................ccgauccgugagcucagc..................................................................................................................................................................................   2    0   LCK-3
.......................ccgauccgugagcucagc..................................................................................................................................................................................   4    0   LCK-1
.......................ccgauccgugagcucagc..................................................................................................................................................................................   4    0   JT-3
.......................cNgauccgugagcucagc..................................................................................................................................................................................   1    1   JT-1
.......................ccgauccgugagcucagc..................................................................................................................................................................................   6    0   LT-3
.......................ccgauccgugagcucagc..................................................................................................................................................................................   3    0   LT-2
.......................ccgauccgugagcucagc..................................................................................................................................................................................   5    0   JCK-1
.......................ccgauccgugagcucagc..................................................................................................................................................................................   2    0   LCK-2
.......................ccgauccgugagcucagc..................................................................................................................................................................................   17   0   JT-1
.......................ccgauccgugagcucagc..................................................................................................................................................................................   2    0   LT-1
.......................ccgauccgugagcucagcuu................................................................................................................................................................................   1    0   JCK-3
.......................ccgauccgugagcucagcuu................................................................................................................................................................................   2    0   LT-2
.......................ccgauccgugagcucagcuu................................................................................................................................................................................   1    0   JCK-1
.......................ccgauccgugagcucagcuuC...............................................................................................................................................................................   1    1   JCK-3
.......................ccgauccgugagcucagcuug...............................................................................................................................................................................   5    0   JCK-2
.......................ccgauccgugagcucagcuuA...............................................................................................................................................................................   1    1   JCK-2
.......................ccgauccgugagcucagcuug...............................................................................................................................................................................   22   0   LCK-1
.......................ccgauccgugagcucagcuug...............................................................................................................................................................................   6    0   JCK-1
.......................ccgauccgugagcucagcuug...............................................................................................................................................................................   26   0   LCK-2
.......................ccgauccgugagcucagcuug...............................................................................................................................................................................   3    0   JCK-3
.......................ccgauccgugagcucagcuug...............................................................................................................................................................................   3    0   LT-2
.......................ccgauccgugagcucagcuug...............................................................................................................................................................................   1    0   JT-2
.......................ccgauccgugagcucagcuuC...............................................................................................................................................................................   2    1   JCK-2
.......................ccgauccgugagcucagcuug...............................................................................................................................................................................   4    0   JT-3
.......................ccgauccgugagcucagcuugu..............................................................................................................................................................................   1    0   JCK-2
.......................ccgauccgugagcucagcuuguuggc..........................................................................................................................................................................   10   0   LCK-1
.......................ccgauccgugagcucagcuuguuggc..........................................................................................................................................................................   14   0   LCK-2
........................cgauccgugagcucagcu.................................................................................................................................................................................   1    0   LT-3
........................cgauccgugagcucagcuu................................................................................................................................................................................   3    0   LT-3
........................cgauccgugagcucagcuug...............................................................................................................................................................................   2    0   LT-1
........................cgauccgugagcucagcuug...............................................................................................................................................................................   2    0   JCK-1
........................cgauccgugagcucagcuug...............................................................................................................................................................................   1    0   JCK-3
........................cgauccgugagcucagcuug...............................................................................................................................................................................   3    0   LCK-3
........................cgauccgugagcucagcuugu..............................................................................................................................................................................   4    0   JCK-2
........................cgauccgugagcucagcuugu..............................................................................................................................................................................   1    0   JCK-3
........................cgauccgugagcucagcuuguu.............................................................................................................................................................................   3    0   LT-1
..........................auccgugagcucagcuugu..............................................................................................................................................................................   1    0   LT-3
..........................auccgugagcucagcuugu..............................................................................................................................................................................   1    0   LT-2
```

```
                    Mature                                Star
uuguucauuuaguaacguauuuaccgauccgugagcucagcuuguuggcauugcacgccguuccuaguugcaggucuucauugcucgguaaaccaaaaaauagcaauguauuuuauuaauuuuauuugauaaaauuuauuaauaucacuuuugaugagacauaaauuaaacaagucuauuuaauuagauuuuugacaauaaauugucaauuaauuaacuuuugacaacuugcauauauuugucguuguac
..............................gugagcucagcuuguuggcauugca...................................................................................................................................................................................................	1	0	JCK-2
...............................ugagcucGgcuuguuggcauugcac..................................................................................................................................................................................................	1	1	LT-3
```
